# Supplementary material for: River plastic emissions to the world's oceans
Source: Nat Commun. 2017 Jun 7;8:15611. doi: 10.1038/ncomms15611 (PMC5467230; doi:10.1038/ncomms15611)
Supplement: Supplementary Information — Supplementary Table and Supplementary References [file ncomms15611-s1.pdf]

Supplementary Table 1: Sensitivity analyses of river plastic size distribution and particle mass variations on the global plastic input estimate. As not all observational studies considered in this assessment reported mass concentration and/or macro-plastics numerical concentration (Table 2), a data homogenisation effort required extrapolations from micro-plastics numerical concentration (Table 3). The parameters used for the homogenisation of our dataset are the average ratio between macro- and micro-plastic numerical concentrations, the average mass of micro-plastics and the average mass of macro-plastics. Ranges of values for these three parameters were based on reported values for micro-plastics and macro-plastics in rivers [1] [2] and at sea [3]: respectively 0.04 (range: 0.01 – 0.12), 0.003 g (range: 0.002-0.004 g) and 0.17 g (range: 0.04 – 0.33 g). Results of regression analyses for 27 triplet configurations with coefficient of determination r^2 and parameters *k* and *a* in Equation (1) of main manuscript is compared against resulting global mass input estimates expressed in million tonnes. Lines in bold corresponds to the midpoint, lower and upper estimates presented in this study.

Conc. ratio macro/micro	Mass micro-plastic (g)	Mass macro-plastic (g)	r^2	*a*	*k*	Global input (million tonnes)
**0.04**	**0.003**	**0.17**	**0.93**	**1.52105031**	**0.00185020**	**1.41**
0.01	0.003	0.17	0.93	1.61673329	0.00111442	1.31
0.12	0.003	0.17	0.92	1.45687558	0.00311138	1.85
0.04	0.002	0.17	0.93	1.51803377	0.00180482	1.33
**0.01**	**0.002**	**0.17**	**0.93**	**1.60918960**	**0.00106575**	**1.15**
0.12	0.002	0.17	0.92	1.45512154	0.00308642	1.81
0.04	0.004	0.17	0.93	1.52520604	0.00187579	1.49
0.01	0.004	0.17	0.94	1.62338863	0.00115535	1.45
0.12	0.004	0.17	0.92	1.45858308	0.00313543	1.90
0.04	0.003	0.04	0.93	1.62100922	0.00109515	1.31
0.01	0.003	0.04	0.94	1.71262467	0.00080989	1.55
0.12	0.003	0.04	0.93	1.54461284	0.00159398	1.34
0.04	0.002	0.04	0.93	1.61327987	0.00104620	1.15
0.01	0.002	0.04	0.94	1.70108868	0.00075967	1.28
0.12	0.002	0.04	0.93	1.54007270	0.00155330	1.25
0.04	0.004	0.04	0.94	1.62780715	0.00113616	1.46
0.01	0.004	0.04	0.94	1.72216731	0.00084939	1.80
0.12	0.004	0.04	0.93	1.54882800	0.00163058	1.44
0.04	0.003	0.33	0.93	1.48110907	0.00249943	1.63
0.01	0.003	0.33	0.93	1.57016444	0.00138289	1.30
0.12	0.003	0.33	0.91	1.42399283	0.00444442	2.37
0.04	0.002	0.33	0.93	1.47871788	0.00246983	1.57
0.01	0.002	0.33	0.93	1.56460400	0.00133868	1.19
0.12	0.002	0.33	0.91	1.42292941	0.00442572	2.34
0.04	0.004	0.33	0.93	1.48341195	0.00252752	1.68
0.01	0.004	0.33	0.93	1.57523773	0.00142171	1.41
**0.12**	**0.004**	**0.33**	**0.91**	**1.42503986**	**0.00446273**	**2.41**

Supplementary References

[1] Van der Wal, M. et al. Identification and Assessment of Riverine Input of (Marine) Litter. Final Report for the European Commission DG Environment under Framework Contract No ENV.D.2/FRA/2012/0025 (2015).

[2] Lechner, A. et al. The Danube so colourful: A potpourri of plastic litter outnumbers fish larvae in Europe's second largest river. Environ. Pollut., **188**, 177-181 (2014).

[3] Eriksen, M. *et al.* Plastic pollution in the world's oceans: more than 5 trillion plastic pieces weighing over 250,000 tons afloat at sea. *Plos One* **9,** e111913 (2014).
